# Auscultation, Percussion and Urinalysis

**Published:** 1884-07

**Authors:** 


**Auscultation, Percussion and Urinalysis**: An Epitome of the Physical Signs of the Diseases of the Heart, Lungs, Liver and Kidneys. Edited by C. Henri Leonard, M. A., M. D., Professor of the Medical and Surgical Diseases of Women, and Clinical Gynæcology, Michigan College of Medicine. Fully illustrated. Cloth, 16mo., 166 pages, post-paid $1.00. Detroit, Mich.: The Illustrated Medical Journal Co., Publishers, 1884.

This little book covers a great deal of ground. It has no less than 163 pages one-half the size of this, and the perusal of the following list of its contents will satisfy the reader as to its value:

Contents—Chap. I.—Topography of the Chest, Anterior and Posterior. Chap. II.—The Physical Diagnosis of Diseases of the Respiratory Organs. Chap. III.—Diagnosis by Percussion. Percussion in Health and Disease. Chap. IV.—Auscultation of the Chest in Health and Disease; also of Voice, Cough and different Rales. Chap. V.—On the Sputa, Microscopical and Macroscopical, with a brief Histology of Lung Structure. Chap. VI.—Diseases of the Lungs; their Pathology and means for Physical Diagnosis. Chap. VII.—On the Pulse; its Rate, Rythm and Sphygmography. Chap. VIII.—The Heart; its Regional Anatomy, Area of Dullness on Percussion in Health and Disease. Chap. IX.—Auscultation of the Heart; the different Cardiac Murmurs and their Indications of Disease. Chap. X.—Diseases of the Heart; their Pathology and Physical Signs. Chap. XI.—The Liver; its Regional Anatomy, Histology, and Physical Signs of the different Diseases. Chap. XII.—The Spleen; its Regional Anatomy, Histology, and Physical Signs of Disease. Chap. XIII.—The Kidney; its Regional Anatomy, Histology, Pathology, and Symptoms of different Diseases. Chap. XIV.—Urinalysis, Chemical and Microscopical; prepared specially for this work by William H. Rouse, M. D., Ph. C. Chap. XV.—Bacteria, Bacilli, Micrococci, Vibriones, and Spirillæ; their Growth, Microscopy, and Agents destructive to them.

